# Supplementary material for: Preconception care utilization: Self-report versus claims-based measures among women with Medicaid
Source: PLOS Glob Public Health. 2023 Nov 30;3(11):e0002592. doi: 10.1371/journal.pgph.0002592 (PMC10688716; doi:10.1371/journal.pgph.0002592)
Supplement: Attachment — Submitted filename: PLOS GPH response letter_19OCT23.docx [file pgph.0002592.s001.docx]

**Author Response Letter**

We are grateful for this review and have responded to all instructions and comments in detail, as described below. We list line numbers where we have made a change in the text. These line numbers correspond to the Revised Manuscript with Track Changes. We have also made minor edits throughout the manuscript, for the sake of consistency and clarity, which we summarize at the bottom of this letter.

Journal Instructions

1. Please review your reference list to ensure that it is complete and correct. If you have cited papers that have been retracted, please include the rationale for doing so in the manuscript text, or remove these references and replace them with relevant current references. Any changes to the reference list should be mentioned in the rebuttal letter that accompanies your revised manuscript. If you need to cite a retracted article, indicate the article’s retracted status in the References list and also include a citation and full reference for the retraction notice.

AUTHOR RESPONSE: We have reviewed the reference list and confirmed it contains no retracted papers.

2. Please provide separate figure files in .tif or .eps format only and remove any figures embedded in your manuscript file. Please also ensure all files are under our size limit of 10MB.

AUTHOR RESPONSE: We have provided figures in .tif format.

3. Potential Copyright Issues:

Fig 1: please (a) provide a direct link to the base layer of the map (i.e., the country or region border shape) and ensure this is also included in the figure legend; and (b) provide a link to the terms of use / license information for the base layer image or shapefile.

AUTHOR RESPONSE: The base layer of the map comes from the United States Census Bureau and therefore is not subject to copyright. We have added this to the legend of the map (Fig. 2).

(a) Source: <https://www.census.gov/geographies/mapping-files/time-series/geo/carto-boundary-file.html>

(b) Terms of use: <https://www.census.gov/data/software/x13as/disclaimer.html>

Additional Editor Comments:
1. Please include 95% CI for the proportion estimates in the abstract --to indicate the difference in the two groups.

AUTHOR RESPONSE: We have added this for the preconception care proportion estimate from PRAMS and have added wording to clarify this is an estimate (see line 34). The proportion in MAX is not an estimate but a direct observation of the population proportion, so a confidence interval is not appropriate.

2. Was the association between variables adjusted for confounders? How was handled?

AUTHOR RESPONSE: Yes, we identified and adjusted for the following confounders available in data files: age, race and ethnicity, and the presence of relevant chronic conditions (diabetes, hypertension and depression). All were included as covariates in the logistic regression models reported in Tables 3 and 4, including differences between states (Table 3). Thus, the reported associations between each variable and the receipt of preconception care may be interpreted as being conditional on the other variables included in the model. We have clarified this in the abstract (line 35) and described it in further detail in the manuscript text (lines 162-165).

3. The reported OR is it adjusted or not?

AUTHOR RESPONSE: Yes, Tables 3, 4, and 5 present odds ratios adjusted for all covariates shown in the table. We have clarified this in the abstract (line 37–8) and the manuscript’s Results section (line 205) as well as the Table 3 header that mentions odds ratios.

4. What are the limitations of this study?

AUTHOR RESPONSE: We have clarified where in the Discussion (lines 258 and 276) we mention study limitations, including: lack of information on many important disease and psychosocial states, lack of information in MAX on preconception services received outside of Medicaid coverage, and potential quality variation in race and ethnicity identification in MAX files.

Reviewer 1:

This is a great paper covering a very important topic, especially on some marginalized populations in the States. The authors have done a terrific job putting the paper together in a good academic fashion with good written English. However, there are some comments I would like to make.

1. Abstract: under the conclusion subheading. This summary is unclear. Can you add a bit more detail, just a short one

AUTHOR RESPONSE: We have reworded the Conclusion section of the abstract and added more detail.

2. Line 208, fix the verb “was”

AUTHOR RESPONSE: We are not sure what the reviewer is requesting here. The sentence in question reads: “In MAX, the standard deviation (SD) of the state-level random effects in the model for all preconception care domains was 0.32 (95% CI 0.25–0.42), corresponding to only 3% of the total variance after adjusting for the covariates.” In other words, the subject of the sentence is “the standard deviation” and the verb is “was,” consistent with all of the other results which are stated in past tense. If necessary, please provide additional detail on the requested change.

3. Discussion: Can you explain why PCU rates in MAX and PRAMS were higher for non-Hispanic Black versus non-Hispanic White women? Give reference for your claims

AUTHOR RESPONSE: We have added further discussion of why this difference may exist, with a reference to support our reasoning. See lines 262-264.

4. Line 35, Discussion: In the previous section, for example, in Table 2, you mentioned Asian and Pacific Women of origin. Is this a different cohort?

AUTHOR RESPONSE: No, all women are from either the MAX claims database or the PRAMS survey. To clarify how we derived these, we have added a sentence in Methods (lines 150-152).

5. Line 51-54, Discussion: Can you summarize your claims with statistics and references?

AUTHOR RESPONSE: The lines in question contained several speculative claims, which we have removed. The remaining statements in the discussion are supported by statistics and references. See lines 268-276.

Reviewer 2:

This is a very important topic for discussion and the authors did a good job with this manuscript. The various components are well written and the available data elaborates what is expected to be seen at the population level.

AUTHOR RESPONSE: Thank you, no changes have been made in response to Reviewer 2.

Other minor edits:

1. We have revised a few uses of abbreviations in order to maximize consistency, for example using the word “Figure” throughout, instead of “Fig” in some places.
2. We have tried to assure that we define each acronym before our first use of it (e.g. PRAMS, PCU, IRB) and then consistently use that acronym throughout the manuscript.
3. A handful of additional copy edits (e.g., removal of unnecessary punctuation marks) are also made throughout. These are all visible in the Revised Manuscript with Track Changes document.
